# Characteristics and requirements of hypertensive patients willing to use digital health tools in the Chinese community: a multicentre cross-sectional survey

**DOI:** 10.1186/s12889-020-09462-2

**Published:** 2020-09-01

**Authors:** Shiqun Chen, Guoli Sun, Xiaolin Cen, Jin Liu, Jianfeng Ye, Jiyan Chen, Li Lei, Yibo He, Feier Song, Wei Guo, Yan Liang, Yuying Hu, Kaihong Chen, Liling Chen, Ning Tan, Yong Liu

**Affiliations:** 1 Department of Cardiology, Guangdong Provincial Key Laboratory of Coronary Heart Disease Prevention, Guangdong Cardiovascular Institute, Guangdong Provincial People’s Hospital, Guangdong Academy of Medical Sciences, Guangzhou, 510000 Guangdong China; 2 Guangdong Pharmaceutical University, Guangzhou, 510006 Guangdong China; 3 Department of Cardiology, Dongguan People’s Hospital, Dongguan, 523000 Guangdong China; 4 Maoming People’s Hospital, Maoming, 525000 Guangdong China; 5 Department of Cardiology, First People’s Hospital of Kashgar, Kashgar, 844099 Xinjiang China; 6 Department of Cardiology, Longyan First Affiliated Hospital of Fujian Medical University, Longyan, 364000 Fujian China

**Keywords:** Digital health tools, Hypertension, Willingness, Community, Blood pressure management

## Abstract

**Background:**

Digital health tools (WeChat or mobile health apps) provide opportunities for new methods of hypertension management for hypertensive patients. However, the willingness of these patients to use social media and mobile health apps for hypertension management remains unclear. This study explored the characteristics and requirements of patients willing to use digital health (WDH) tools to manage hypertension.

**Methods:**

From February to March 2018, we administered questionnaires to 1089 patients with hypertension at eight Chinese primary medical units. We assessed independent risk factors of WDH and requirement among WDH patients.

**Results:**

Overall, 43% (465/1089) of participants were WDH patients, who were younger (58 ± 12 vs 61 ± 13 years) and had a greater proportion of employed individuals (31% vs 14%) and higher education levels (65% vs 52%) than the non-WDH patients (all *P* < 0.0001). After adjusting for other risk factors, higher education (OR: 0.52; 95% CI: 0.34–0.79), good medicine adherence (OR: 1.5; 95% CI: 1.0–2.3) and blood pressure self-monitoring (OR: 1.6; 95% CI: 1.2–2.3) remained significantly associated with WDH (all *P* < 0.05). WDH patients responded that digital health tools should try to provide a platform for blood pressure monitoring (42%), medication reminders (41%), hypertension knowledge (39%) and doctor-patient communication (32%).

**Conclusion:**

Our survey suggested that among hypertensive patients, willingness to use digital health tools was significantly associated with education, medicine adherence and blood pressure self-monitoring. Digital health tool developers and researchers should pay particular attention to recruiting older, less educated and unemployed patients with less willingness and who are less technologically savvy and research the requirements of WDH patients (blood pressure monitoring, medication reminders, and knowledge education) in the future.

## Background

### Status of hypertension

Hypertension has become the leading cause of cardiovascular death due to its low control rate [1] in both low- and middle-income countries around the world [2, 3]. One national study that included 1.7 million participants aged 18 years and older showed that the awareness, treatment, and control rates of hypertension were 31.9, 26.4 and 9.7%, respectively [4]. In addition, the 2018 Hypertension Guidelines recommend that hypertension management, in addition to drug intervention, should focus on lifestyle interventions, home self-tests, follow-up, and the use of smartphones and other kinds of remote monitoring tools for blood pressure [5].

Digital health interventions, such as email, text messages, smartphone applications like facebook, have been explored to assist in the self-management of hypertension and some other chronic diseases [6]. Many trials have proved that mobile health applications played an important role in improving adherence in high-income countries, by providing reminders, offering education, and facilitating social interactions and so on [7–10]. A randomised clinical trial has already showed the efficacy of the social media-based prevention on control of systolic blood pressure and heart rates in China [11, 12]. However, the use and expectations of digital health tools for community patients with hypertension is still unclear. Hence, this survey was conducted to explore 1) the characteristics of patients willing to use digital health tools to manage hypertension (WDH) and 2) the required features of these digital health tools.

## Methods

### Sample and procedure

A sample of 1089 individuals from eight primary medical units in Guangzhou and Dongguan, China, participated in this survey. Data were collected in February and March of 2018. Participants had to meet the following inclusion criteria: (1) community resident with hypertension and (2) age 18 years and older. There were no exclusion criteria. All of the interviewed individuals finished the survey, and the mean time required for participants to finish the survey was 20 min. This study was conducted in compliance with the Declaration of Helsinki; the study was also approved by the Ethics Research Committee of Guangdong Provincial People’s Hospital, and written informed consent was obtained from all patients [13].

### Sociodemographics

Sex, age, height, weight, education (International Standard Classification of Education, ISCED), occupation, and medical care were evaluated by standard survey items. (Supplementary Table 1).

### Hypertension management conditions

To investigate the current status of participants’ management of hypertension, we designed the following questions in our questionnaire. First, questions on the knowledge of the diagnosis of hypertension, when the patient should measure blood pressure, and the damage (heart/brain/kidney) from high blood pressure were asked. Next, patients’ usual systolic blood pressure fluctuation, the frequency of blood pressure measurement and antihypertensive dosing were surveyed to understand participants’ blood pressure monitoring status and medical adherence. Last, the total time of participants’ daily activities [14] and smoking status were also asked to explore the relevance of digital health tool usage to exercising and smoking.

The definition of “good BP monitoring” is that the patient’s blood pressure is under 140/90 mmHg in their daily life. Adherence was assessed by asking participants how often they forget to take medicine. The answers “never” and “less than once a week” were defined as good adherence. The answers “more than one time a week” and “Don’t take medicine when blood pressure is under control” were defined as bad adherence.

### The willingness to use digital health tools

In the final part of the questionnaire, we surveyed participants’ willingness and requirements for the use of digital health tools from the following three questions: (1) Have you used WeChat or health applications in the last 1 year? (2) Would you like to use a digital health tool to manage your blood pressure? (3) What help would you like to get from the health apps (1 week’s blood pressure monitoring situation, one-week medication adherence status, self-blood pressure control evaluation, hypertension knowledge, patient-patient communication, or doctor-patient communication)?

### Statistical analyses

We analysed the entire sample as well as specific subgroups, and we mainly compared WDH patients and non-WDH patients. Binary logistic regression models were applied. Baseline characteristics of the cohort were summarized using descriptive statistics. Continuous variables were reported as the mean and SD and were compared using linear regression models. Potential confounders and baseline values of the dependent variables were entered as covariates. Categorical variables were described as frequencies and percentages and compared using the χ2 test. The criterion for statistical significance was set at *p* < 0.05. The statistical analysis was conducted using SAS V.9.4.

## Results

### Characterization of the sample

The total number of people participating in this community survey was 1089 (see Table 1), with an average age of 60 (±13) and a mean BMI of 24.5 (±4.3); 53.9% were males. It was found that 79.0% of the patients had no more than a junior high school education.

**Table 1:** Sample characteristics of WDH patients

Item	Total sample	WDH	No WDH
	100% (1089/1089)	42.7% (465/1089)	57.3% (506/1089)
	*N* = 1089	*N* = 465	*N* = 624
Male (vs female), n (%)	549 (50.4)	218 (60.3)	331 (39.7)
Age (SD)	60 (13)	58 (12)	61 (13)
Age > 75 years, n (%)	160 (15.4)	112 (24.1)	48 (7.7)
BMI (SD)	24.5 (4.3)	24.45 (4.8)	24.48 (3.9)
Educational level, n (%)			
High school or above	816 (79)	286 (35.1)	530 (64.9)
Junior high school or less	217 (21)	129 (59.5)	88 (40.5)
Employment status, n (%)			
working	590 (57.2)	270 (45.8)	320 (54.2)
not working	441 (42.8)	144 (32.6)	297 (67.4)
Medical insurance, n (%)			
medical care	916 (89.5)	362 (39.5)	554 (60.5)
no medical care	108 (10.5)	47 (43.5)	61 (57.5)
Mean SBP fluctuation, n (%)			
< 140 mmHg	587 (57.6)	227 (38.7)	360 (61.3)
≥ 140 mmHg	432 (42.4)	176 (40.7)	256 (59.3)
Knowledge of diagnostic criteria, n (%)			
≥ 140/90 mmHg	501 (46)	224 (44.7)	227 (55.3)
other	588 (54)	241 (41)	347 (59)
Acknowledgement of complications of hypertension (diseases caused by hypertension), n (%)			
myocardial infarction	528 (51.3)	267 (50.6)	261 (49.4)
stroke	529 (51.5)	270 (51)	259 (49)
renal impairment	470 (45.8)	247 (52.6)	223 (47.4)
BP monitoring, n (%)			
good BP monitoring	564 (51.8)	243 (43.1)	321 (56.9)
poor BP monitoring	525 (48.2)	222 (42.9)	303 (57.7)
Medical adherence, n (%)			
good adherence	801 (73.5)	326 (40.7)	475 (59.3)
poor adherence	288 (26.5)	139 (48.3)	149 (51.7)
Smoking, n (%)			
never smoked or quit	836 (76.8)	367 (43.9)	469 (56.1)
smoked	253 (23.2)	98 (38.7)	155 (61.3)
Physical activity, n (%)			
weekly high-intensity exercise	641 (63)	262 (40.9)	379 (59.1)
absence of the above exercise intensity	376 (37)	137 (36.4)	239 (63.6)

Almost half of participants were able to recognize the diagnostic criterion of hypertension and complications, and measure blood pressure once a month at least. Although 73.6% patients had a good medical adherence, only half patients could control their blood pressure under 140/90 mmHg (57.6%). Majority of participants have a good habit in smoking (76.8%) and exercise (63.0%). Out of all participants, 42.7% reported that they were willing to use digital health tools to manage hypertension.

### Risk factors for WDH patients

The results from binary logistic regression (see Table 2) revealed that WDH patients were more likely to have a higher education level (OR: 1.92; 95% CI: 1.27–2.94, *P* < 0.05) and that they were more likely to have good medicine adherence (OR: 1.5; 95% CI: 1.0–2.3, *P* < 0.05) and blood pressure self-monitoring (OR: 1.6; 95% CI: 1.2–2.3, *P* < 0.05). WDH was not associated with sex, age, BMI, marriage status, working time, knowledge of hypertension, SBP fluctuation, or the health behaviours of smoking and physical activity. Differences of baseline between patients with controlled hypertension and uncontrolled hypertension can be found in the Supplement Table 2.

**Table 2:** Multivariate associations with WDH patients ^a-b^

Item	WDH ^a^		
	Odds Ratio	95% CI	*P*
Male (vs female)	1.17	0.83–1.65	0.36
Age	0.99	0.98–1.01	0.62
BMI	1.00	0.97–1.04	0.90
Marriage vs no marriage	1.14	0.73–1.78	0.56
Good education vs Poor education	1.92 ^b^	1.27–2.94	0.002
Unemployment vs employment	0.72	0.47–1.10	0.13
Medical insurance vs no medical insurance	1.04	0.61–1.76	0.90
Hypertension diagnosis	1.21	0.87–1.70	0.26
Usual SBP fluctuation< 140 mmHg vs ≥140 mmHg	1.11	0.78–1.59	0.56
Good BP monitoring vs poor BP monitoring	1.64 ^b^	1.19–2.26	0.002
Good medical adherence vs poor medical adherence	1.52 ^b^	1.01–2.28	0.044
Smoking vs no smoking	0.79	0.53–1.16	0.23
Less physical activity vs more physical activity	1.04	0.74–1.47	0.82

^a^WDH refers to non-WDH

^b^Still significant after correction for multiplicity

### Requirements of WDH patients for digital health tools

When WDH patients were asked about what help they would like to obtain from digital health tools, a significant portion of participants (42%) indicated that they were eager to engage in one-week blood pressure monitoring, and a similar percentage (41%) wanted feedback on 1 week of medical adherence. Thirty-nine percent of WDH patients wished to obtain some simple knowledge of hypertension, and 32% of them needed doctor-patient communication. In addition, 28% of WDH patients thought that self-control of blood pressure was needed, and 27% reported that they would like to communicate with other patients.

## Discussion

This is a relatively new cross-sectional survey on the willingness of patients with hypertension to use digital health tools, which covered 1089 people with hypertension from different economic backgrounds, revealing a certain representativeness. Our survey found that 42.7% of hypertensive patients were willing to use digital health tools, and WDH was associated with higher education, good medicine adherence and blood pressure self-monitoring. Also, the results showed that most WDH patients wanted to receive scientific blood pressure monitoring, self-administration of medical adherence and common knowledge about hypertension.

We searched a large number of articles about social media for health care, which show the organic combination of social media and medical health. A systematic review reviewed 45 social media use improvements in chronic disease management, showing that social media, especially Facebook and blogs, provides social, spiritual, and empirical support for chronic diseases. These platforms are thus highly likely to improve patient health [15]. Research by Julie Redfern of the George Institute of Global Health in Australia found that social media (Tweets) can disseminate cardiovascular health information and education quickly, efficiently and globally, and discovered that social media has great potential to promote cardiovascular disease education, cognition and comprehensive management [16]. Facebook, as a social media platform, was also found to have a good auxiliary function for clinical treatment of cardiovascular disease [17, 18]. Social media were widely used in chronic diseases and clinical research, but there are few studies on self-management of patients with hypertension [19, 20]. Secondly, the social media platforms which are currently being applied and studied are mainly based on popular software (such as Facebook, Tweets, SMS, etc.) in western countries. To the best of our knowledge, this is the first study to explore the willingness of using local social media as a health tool in China. We can assume that WeChat will provide an advanced and intelligent way for self-management of hypertension patients in the community.

In a recent longitudinal survey, Levine et al. [21] found that older people with an average age of 75 used digital health at a lower rate than their younger counterparts, but this rate increased modestly from 2011 to 2014, highlighting the rising importance of mobile technology in people’s lives. A population-based survey of the use of a national smartphone and health apps among Germans in 2017 also identified age as a correlate for mobile health applications use: younger people were more engaged [22], and our study confirmed this for hypertensive study population as well. In addition, our results contribute to previous findings on literacy-related disparities in access to mobile technologies by revealing an association between WDH and education level [23]. Therefore, when age and literacy levels are correlated with WDH, programmers should consider the needs of older and less educated people when developing applications. Numerous efforts should be made in the future to break the rigid relationship between ageing and the digital gap to make digital technology more acceptable and easier to use.

We contributed to the preliminary evidence of the correlation of WDH with blood pressure self-management and medical adherence. Patients with poor blood pressure management and medical adherence are relatively reluctant to use digital health tools, which may be the result of a combination of factors such as age, smartphone maturity, personal income, tool trust, and so on. The relationship between blood pressure management and WDH may be explained by socioeconomic variables. Indeed, we found that possession of university degrees and employment were related to the use of WeChat and mobile health tools, and further research on this correlation is needed. More studies are needed on the features of effective digital health tool programs. Social media and health application effectiveness information based on evidences from rigorous research designs should be provided to users. Given the sheer number of applications available, the market transparency will be increased. Not only government guidelines and regulations, but also WHO recommendations could help people choose effective and appropriate applications [24]. The first attempt in this regard was directed at the MEDDEV guidelines 2.1/6 for the European market and the US Food and Drug Administration (FDA) guidelines for the US market [25, 26]. While these are not legally binding, they provide a direction for developers and consumers of mobile health applications. Furthermore, as the features develop, whether social media can be regarded as a newly integrated digital health tool and replace the function-pure applications, needs more trials to prove conclusively.

We found differences in age and literacy rates in the use of mobile technology. Therefore, application developers and researchers should consider the needs of older people and those with lower education levels, for example, to provide customized features that are tested in intervention studies [16, 24]. Simultaneously, advocacy activities should be carried out to train older people to use mobile technology, improve the health literacy of the population, and reduce the inequalities resulting from technological advances.

### Limitation

Due to the cross-sectional character of the survey, changes could not be checked, including associations of digital health tools and their characteristics with actual blood pressure and behavior management. The patient’s economic situation has not yet been included in the questionnaire, making the association of income with blood pressure management and digital health tools impossible to determine at this moment. Previous studies have found that the use of apps is associated with higher incomes [27]. Another aspect is that the characteristic of the questionnaire limited the conclusions that could be drawn from the results. For example, BMI was calculated based on self-reported weight and height which is a possible source of bias. The study enrolled patients in hospitals and communities. When there is a significant difference in admission rate between hospitals communities, admission rate bias is unavoidable.

Furthermore, a large number of analyses were conducted which could have increased the probability of type I errors (ie, stating an effect when none was present). However, our research is exploratory. Nonetheless, in addition to the uncorrected results, we decided to report the multiplicity corrected results following a recommendation by Streiner [28], who discussed arguments for and against a correction of multiplicity. Namely, our ambition involves providing a large informational basis that can be further examined in future research.

## Conclusion

Our survey suggested that among patients with hypertension, willingness to use digital health tools was significantly associated with higher education, good medicine adherence and blood pressure self-monitoring. In addition, we need to focus on WDH patients’ requirements for blood pressure monitoring, medication reminders, and hypertension education to improve the technology of digital health tools and research in the future.

## Supplementary information

**Additional file 1: Table S1.** Questionnaire for community hypertension patients (English language version and original version).

**Additional file 2: Table S2.** Differences of baseline between patients with controlled hypertension and uncontrolled hypertension.

## Data Availability

The datasets generated and analysed during the current study are not publicly available due the institution policy but are available from the corresponding author on reasonable request.

## Abbreviations

WDH: Willing to use Digital health tools to manage Hypertension
ISCED: International Standard Classification of Education
RESCIND: The Center for REduction of contraSt-induced nephropathy and Cardiaovascular events followINg carDiac catheterization
GDPH: Guangdong Provincial People’s Hospital
BMI: Body mass index
BP: Blood pressure
SBP: Systolic blood pressure
WHO: World Health Organization
FDA: Food and Drug Administration
