# Supplementary material for: Characteristics and requirements of hypertensive patients willing to use digital health tools in the Chinese community: a multicentre cross-sectional survey
Source: BMC Public Health. 2020 Sep 1;20:1333. doi: 10.1186/s12889-020-09462-2 (PMC7465797; doi:10.1186/s12889-020-09462-2)
Supplement: Supplementary file 1 — Additional file 1: Table S1. Questionnaire for community hypertension patients (English language version and original version). [file 12889_2020_9462_MOESM1_ESM.docx]

**English language version**

Questionnaire for community hypertension patients

Part 1: Personal information

1. Your gender is:

A male B Female

1. Your date of birth is
2. Your height is cm, and your weight is cm.
3. Your cultural education level is:

A elementary school and below B junior high school

C high school D university and above

1. Your job:

A full time job B part time job C retirement

D no job E student at school

Part 2: Hypertension management conditions

1. What do you think is the value of blood pressure to diagnose hypertension in Chinese adults? A Unclear

B Systolic blood pressure ≥ 150mmHg and or diastolic blood pressure ≥ 90 mmHg

C Systolic blood pressure ≥ 140mmHg and or diastolic blood pressure ≥ 90 mmHg

1. Do you think that hypertension can cause myocardial infarction, stroke or renal impairment ? A Yes B No C Don't know
2. What is your blood pressure situation?

A measure at home, but not self-adjusting

B measure at home and adjusting the drug according to blood pressure value

C measure only in the hospital office

1. Please answer the following questions about medication with “Yes” or “no”.
2. Did you take the medicine yesterday?
3. Have you forgotten to take the medicine in the past two weeks?
4. Did you forget to take the medicine?
5. When you feel that your symptoms are worse or other discomfort, do you increase your dose or change your medication?
6. Have you ever forgotten to take your medicine with you when you are traveling or leaving home for a long time?
7. When you feel that your blood pressure has been controlled, have you stopped taking the medicine?
8. Is it difficult for you to take medication and measure blood pressure as required?
9. Did you forget the time and amount of medication?

5、Your smoking status is:

A Never smoke B Still smoking C Smoking in the past and quit now

6、You drinking habit is:

A Drinking a large amount (an average of 1 white liquor per day, or a relatively alcoholic wine or beer)

B Drinking a small amount (less than A daily alcohol consumption)

C Never drinking

7、The recent physical activity situation is:

A High-intensity exercise weekly (similar to long-distance running, football, etc.) ≥ 3 times or moderate-intensity exercise (similar to brisk walking, swimming, playing volleyball, etc.) ≥ 3 times, each time more than 30 minutes.

B Didn't reach the above exercise intensity/frequency

8、Is your diet balanced?

A Balance (each with vegetables, fruits and grains)

B Unbalanced

9、You think that your salt intake is:

A Salty: Daily salt intake per person ≥ 6 grams (one beer bottle cap *), pay attention to the intake of hidden salt (salty, chicken, soy sauce, etc.)

B Less salt (not meeting the above criteria)

10、Do you think you are a low-fat diet (rarely eat animal fat, fried foods, junk food, etc.)?

A Yes B No.

Part 3: The use of WeChat and mobile health apps

1. Have you used WeChat in the past year? Yes or No
2. Have you used health applications (apps, applets) in the past year? Yes or No
3. What health behaviors do you want to promote by health applications?

A Quitting smoking B exercise C weight loss D balanced diet

E alcohol withdrawal F others

1. What features does the health application you use have?

A provision of information on the target behavior

B motivational messages

C goal setting and action planning

D Reminder

E information on the current status and individual progress

F I do not use any of these app characteristics

1. Are you willing to use the hypertension management application?
2. What is your reason for using the hypertension management application?

A recommendation by doctors and nurses

B recommendation by relatives and friends

C good reputation

D free

E not applicable

F simple and convenient to use

G others

1. What help would you like to get from the health apps?

A One week's blood pressure trend

B one week medication

C self-blood pressure control evaluation

D hypertension knowledge

E patients’ communication

F communication with doctor if necessary

**original version**

《高血压患者问卷调查》

一、个人信息：

1.您的性别是:? A.男 B.女

2.您的出生年月是: ________年_______月_______日

3.您的身高：______________cm 体重：______________kg

4.您的文化教育程度是?

A.小学及以下 B.初中 C.高中 D.大学及以上

5.您每天的工作时长是?

A.全职工作 B.兼职工作 C.退休 D.不工作 E 在校学生

二． 高血压个人情况

1.您认为中国成人血压高于多少被诊断为高血压?

A.不清楚

B.收缩压≥150mmHg和或舒张压≥90 mmHg

C.收缩压≥140mmHg和或舒张压≥90 mmHg

2.您认为高血压会引起心梗，卒中，肾功能损害吗？

A.会 B.不会 C.不知道

3.您测血压（自我血压监测与管理）情况是？

A.在家自己测血压,但不会自己调药

B.在家测血压并根据血压值调整药物

C.仅在医院诊室测血压

4.请回答以下降压药服用情况？（打勾√）

a)昨天您服药了吗？ 是（ ） 否（ ）

b)在过去的２周内，是否有忘记服药的情况？ 是（ ） 否（ ）

c)您是否会忘记服药？ 是（ ） 否（ ）

d)当您觉得症状加重或出现其他不舒服时，您是否会自行加大药量或更改物？ 是（ ） 否（ ）

e)当您外出旅行或长时间离家时，您是否曾经忘记随身携带药物？

是（ ） 否（ ）

f)当您觉得血压已经得到控制时，是否停止过服药？ 是（ ） 否（ ）

g)坚持按要求服药和测量血压对您来说是否有困难？ 是（ ） 否（ ）

h)您是否会忘记服药时间和服药量？

从不（ ） 偶尔（ ） 有时 （ ） 经常（ ） 所有时间（ ）

5.您的吸烟状况?

A.从不抽烟

B.近期仍在抽烟

C.过去抽烟，现在已戒烟

6.您的饮酒习惯是?

A.长期饮酒（平均每日1两白酒，或相当酒精含量的葡萄酒或者啤酒）

B.有饮酒，但量少（少于A选项的每日饮酒量）

C.从不饮酒

7.最近运动情况是？

A．每周高强度运动（类似长跑、踢足球等运动）≥3次或者中等强度运动（类似快步走、游泳、打排球等运动）≥3次，每次30分钟以上。

B．没有达到以上运动强度/频次

8.您认为您的饮食均衡吗?

A.均衡（每顿含蔬菜，水果和谷物）

B.不均衡

9.您认为您摄入盐的情况是？

A.偏咸：每人每日食盐摄入量≥ 6 克（一啤酒瓶盖 *），注意隐性盐的摄入

（咸菜、鸡精、酱油等）

B.少盐清淡（没有达到以上标准）

10.您认为您是低脂饮食（很少吃动物脂肪、油炸食品和垃圾食品等）吗?

A.是 B.不是

三．互联网医疗使用状况

1.那您过去1年内是否使用过微信?

A.否 B.是

2.那您过去1年内是否使用过健康应用软件?（APP，小程序）

A.否 B.是

3.那您使用健康应用软件是为了促进以下哪些健康行为？（可多选）

A.戒烟 B. 运动 C.减肥 D. 健康饮食 E. 戒酒 F其他

4.那您使用过的健康应用软件是否具备以下功能？（可多选）

A.知识推送（养身知识、降压、运动、减肥、健康饮食知识等）

B.激发的积极性信息（类似运动步数、血压、减肥排名或激励）

C.目标设定与行动计划（类似降压、减肥、运动的目标与计划）

D.提醒功能（类似提醒服药、测量血压、运动、低盐饮食及戒烟等）

E.近况与进展（类似降压趋势、减肥进展、运动趋势等）

F.其他

5.为了让医生更好的关注、管理、控制您的血压，您是否愿意使用高血压管理应用软件？

A.已经在使用 B.愿意 C.不愿意

6.您希望高血压管理软件可以改进以下那些健康行为？（可多选）

| A.戒烟 | B.运动 | C.减肥. |
| --- | --- | --- |
| D.健康饮食 | E.规律药物 | F.戒酒 |

G.其他

7._________________________________您希望高血压管理软件有哪些功能（可多选）

A.戒烟 B.运动 C.减肥 D.健康饮食 E.戒酒 F.其他
