# Supplementary material for: Characteristics and requirements of hypertensive patients willing to use digital health tools in the Chinese community: a multicentre cross-sectional survey
Source: BMC Public Health. 2020 Sep 1;20:1333. doi: 10.1186/s12889-020-09462-2 (PMC7465797; doi:10.1186/s12889-020-09462-2)
Supplement: Supplementary file 2 — Additional file 2: Table S2. Differences of baseline between patients with controlled hypertension and uncontrolled hypertension. [file 12889_2020_9462_MOESM2_ESM.docx]

Supplement table 1. Differences of baseline between patients with controlled hypertension and uncontrolled hypertension

| **Item** | Total | Sbp<=140 | Sbp>140 | *P* |
| --- | --- | --- | --- | --- |
|  | N=1039 | N=587（56.5） | N=452（43.5） |  |
| Age（y） (SD) | 61（13） | 62（13） | 59（12） | <0.001 |
| Age>=75 n(%) | 161(15.83) | 106(18.47) | 55(12.42) | 0.009 |
| Male n (%) | 549（53.9） | 303（53.06） | 246（55.03） | 0.53 |
| BMI (SD) | 24.3（3.2） | 24.4（3.1） | 24.1（3.4） | 0.092 |
| Good Education, n (%) | 217(21.0) | 105(18.0) | 112(24.8) | 0.008 |
| Work, n (%) | 451(43.41) | 220(37.5) | 231(51.1) | <0.001 |
| No medical insurance n (%) | 108(10.5) | 29(5.0) | 79(17.6) | <0.001 |
| Knowledge of diagnostic criterion (%) | 613(59) | 427(72.7) | 186（41.2) | <0.001 |
| Acknowledge of complications of hypertension n (%) | 494（47.6） | 352（60.0） | 142（31.4） | <0.001 |
| Good BP monitoring, n (%) | 387(37.25) | 223(38.0) | 164(36.3) | 0.57 |
| Good medical adherence, n (%) | 325（31.4） | 222（38.0） | 103（22.9） | <0.001 |
| Smoking，n (%) | 395(39.5) | 186(32.9) | 209(40.1) | <0.001 |
| Weekly high-intensity exercise, n (%) | 641(61.7) | 328(55.9) | 313(69.3) | <0.001 |
